# Assessment of Erythrocyte Transketolase, Whole Blood Thiamine Diphosphate, and Human Milk Thiamine Concentrations to Identify Infants and Young Children Responding Favorably to Therapeutic Thiamine Administration: Findings from the Lao Thiamine Study, a Prospective Cohort Study

**DOI:** 10.1016/j.cdnut.2024.103786

**Published:** 2024-05-23

**Authors:** Sonja Y Hess, Taryn J Smith, Charles D Arnold, Kerry S Jones, Daniela Hampel, Laurent Hiffler, Indi Trehan, Philip R Fischer, Sarah R Meadows, Damon A Parkington, Kenneth H Brown, Dalaphone Sitthideth, Xiuping Tan, Albert Koulman, Lindsay H Allen, Sengchanh Kounnavong

**Affiliations:** 1 Institute for Global Nutrition and Department of Nutrition, University of California Davis, Davis, CA, United States; 2 Nutritional Biomarker Laboratory, MRC Epidemiology Unit, University of Cambridge, Cambridge, United Kingdom; 3 USDA-Agricultural Research Service Western Human Nutrition Research Center, Davis, CA, United States; 4 Cellular Nutrition Research Group, Lagny sur Marne, France; 5 Departments of Pediatrics, Global Health and Epidemiology, University of Washington, Seattle, WA, United States; 6 Lao Friends Hospital for Children, Luang Prabang, Lao People’s Democratic Republic; 7 Department of Pediatric and Adolescent Medicine, Mayo Clinic, Rochester, MN, United States; 8 Lao Tropical and Public Health Institute, Vientiane, Lao People’s Democratic Republic

**Keywords:** erythrocyte transketolase, thiamine diphosphate, thiamine deficiency, thiamine deficiency disorders, thiamine responsive disorders, beriberi, human milk, Laos

## Abstract

**Background:**

There is limited information on relationships among biomarkers of thiamine status (whole blood thiamine diphosphate [ThDP], erythrocyte transketolase activity coefficient [ETKac], and human milk thiamine [MTh]) and clinical manifestations of thiamine deficiency.

**Objectives:**

This study aimed to explore correlations among these biomarkers and thiamine responsive disorders (TRDs), a diagnosis based on favorable clinical response to thiamine.

**Methods:**

Hospitalized infants and young children (aged 21 d to <18 mo) with respiratory, cardiac, and/or neurological symptoms suggestive of thiamine deficiency were treated with parenteral thiamine (100 mg daily) for ≥3 d alongside other treatments and re-examined systematically. Clinical case reports were reviewed by 3 pediatricians, who determined TRD or non-TRD status. Children in a community comparison group were matched by age, sex, and residence. Venous whole blood ThDP and MTh were determined by high-performance liquid chromatography fluorescence detection and ETKac in washed erythrocytes by ultraviolet spectrophotometry. Associations between biomarkers were assessed using Spearman correlations, and biomarker cutoffs predictive of TRD and ETKac >1.25 were explored using area under the receiver operating characteristic curve framework.

**Results:**

Thiamine biomarkers were available for 287 hospitalized children and 228 community children (mean age 4.7 mo; 59.4% male). Median (interquartile range [IQR]) ThDP and ETKac were 66.9 nmol/L (IQR: 41.4, 96.9 nmol/L) and 1.25 nmol/L (IQR: 1.11, 1.48 nmol/L), respectively, among hospitalized children, and 64.1 nmol/L (IQR: 50.0, 85.3 nmol/L) and 1.22 nmol/L (IQR: 1.12, 1.37 nmol/L) among 228 community children (*P* > 0.05 for both). Forty-five percent of breastfeeding mothers of infants <6 mo had MTh <90 μg/L. ThDP and ETKac, but not MTh, were significantly different between 152 children with TRD and 122 without TRD, but overlapping distributions undermined prediction of individual responses to thiamine.

**Conclusions:**

Although ETKac, ThDP, and MTh are useful biomarkers of population thiamine status, none of the biomarkers reliably identified individual children with TRD. ThDP is more practical for population assessment because preparing washed erythrocytes is not required.

This trial was registered at clinicaltrials.gov as NCT03626337.

## Introduction

Vitamin B_1_ is a family of water-soluble, sulfur-containing compounds essential for human health and development [1]. Thiamine diphosphate (ThDP) is the most abundant form of the vitamin B_1_ family circulating in the blood and is the coenzyme required for several enzymes involved in carbohydrate metabolism and energy production. There is limited information from nationally representative surveys [2]. However, in the 2014 Cambodian National Micronutrient Survey, 27% of women 15 to 49 y of age and 15% of children 6 mo to 5 y of age were thiamine deficient based on a conservative cutoff of erythrocyte ThDP <120 nmol/L, with the highest prevalence of deficiency of 38% among infants aged 6 to 12 mo [3]. Similarly, evidence from smaller studies suggests that thiamine deficiency may be a public health concern in several low- and middle-income countries [[4], [5], [6], [7]]. A national food consumption survey in Lao People’s Democratic Republic (Lao PDR) in 2016–2017 estimated that 85% of pregnant women and 88% of lactating women had insufficient thiamine intakes based on a 24-h dietary recall [8]. Dietary risk factors for thiamine deficiency include the consumption of monotonous diets with high reliance on polished white rice or cassava [9]. Low dietary thiamine intake can be further aggravated by the consumption of thiamine inhibitors (such as betel nut or thiaminase in raw fish) [4], traditional postpartum restrictive diets [[10], [11], [12]], and elevated thiamine requirements during times of increased energy utilization such as acute illness and during pregnancy and lactation [13,14]. Human milk thiamine (MTh) concentrations are related to maternal thiamine intake and status [15], and maternal thiamine deficiency is likely to place the breastfed child at risk of clinical manifestations of thiamine deficiency [9].

Thiamine deficiency can lead to a broad spectrum of clinical abnormalities [16], referred to as thiamine deficiency disorders (TDD), including the classic forms of beriberi in infants and children. The diagnosis of TDD is complicated by the fact that the clinical signs and symptoms associated with TDD overlap with those of other illnesses and/or co-occur with common childhood infections, which can contribute to misdiagnosis. An additional challenge is that several studies found little association between biomarkers of thiamine status and TDD-like symptoms in populations in which thiamine deficiency is common [[17], [18], [19]]. To address this challenge, we recently proposed a predictive model for thiamine responsive disorders (TRD) in an effort to help identify infants and young children who may benefit from thiamine administration [20].

In this study, we explored the relationships among proposed biomarkers of thiamine status with each other and with response to thiamine administration. ThDP can be assessed in either erythrocytes or whole blood as a biomarker of thiamine status [21]. However, there is no consensus on a cutoff for ThDP concentration to determine thiamine deficiency, and a wide range of cutoffs have been used in previous studies [5,22]. ThDP is a coenzyme for erythrocyte transketolase (ETK), and the ETK activity assay can be used as a functional marker to assess the adequacy of thiamine availability [23]. Although there is no international consensus on the ETK activity coefficient (ETKac) cutoff, >1.25 is generally used to define high risk of thiamine deficiency [4,13,22,24]. There is uncertainty about cutoffs to define adequacy of total thiamine concentration in human milk. However, 200 μg/L has been suggested as adequate for the majority of infants in the first half-year of life, and this cutpoint is recommended by the European Food Safety Authority [25].

The objective of the present study was to fill the knowledge gap concerning biomarkers of thiamine status and their associations with clinical manifestations of thiamine deficiency in at-risk populations. Data were derived from the Lao Thiamine Study, a prospective cohort study implemented in northern Lao PDR to develop a case definition for TRD among infants and young children [20,26]. The primary objectives of the present study were to: *1*) evaluate proposed thiamine status biomarkers in relation to clinical signs of deficiency and response to thiamine and explore potential cutoffs for whole blood ThDP, basal ETK activity, ETKac, and MTh; *2*) explore associations between indicators of thiamine status; and *3*) define a population-specific cutoff of ThDP corresponding to ETKac >1.25, separately for young children and women.

## Methods

### Study design, setting, and population

The Lao Thiamine Study was a hospital- and community-based, prospective cohort study in Luang Prabang, Lao PDR, carried out from June 2019 to December 2020. The detailed study protocol has been published previously [26]. Briefly, infants and young children 21 d to <18 mo of age hospitalized at the Lao Friends Hospital for Children with clinical signs or symptoms suggestive of TDD, such as tachycardia, respiratory distress, or altered mental status, were eligible if they met at least one of the inclusion criteria (Supplemental Table S1). Mothers of children enrolled in the hospital cohort were also invited to participate in the study. A community-based comparison group of asymptomatic children and their mothers frequency-matched by residence, age, and sex to the hospitalized children were included in the study for comparison of thiamine biomarkers and potential risk factors for thiamine deficiency (Supplemental Table S1). To achieve a well-balanced match and to avoid seasonal discordance, we characterized the children enrolled in the hospital cohort on a weekly basis.

The Lao Friends Hospital for Children provides free pediatric healthcare serving Luang Prabang and surrounding provinces. Although Luang Prabang is a town of over 90,000 inhabitants and a United Nations Educational, Scientific and Cultural Organization World Heritage site [27], surrounding villages are rural and spread across montane rain forests. The population is predominantly of the Hmong and Khmu ethnic groups, relies on subsistence farming, and is of lower socioeconomic status. The community did not provide input into the study design.

Ethical approval for the study and consenting procedures was provided by the National Ethics Committee for Health Research, Ministry of Health, Lao PDR (ref. 11/2019) and the Institutional Review Board of the University of California Davis (ref. 1329444). Written informed consent was obtained from ≥1 parent or the primary caregiver for the children’s participation, and from the women for their own participation.

### Data collection

Once children were enrolled in the hospital cohort, an extensive baseline health assessment was performed. Four, 8, 12, 24, 36, 48, and 72 h after the first thiamine administration, the physical examination was repeated, the child’s clinical status was assessed using a 5-point scale (seems worse; no change; seems a little bit better; seems a lot better; seems recovered), and whether the child was well enough to be discharged from hospital [26]. Hospital physicians were responsible for treatment of all children in the hospital cohort, and all administered drugs and medical interventions were recorded by the study team along with the hospital physician diagnosis at discharge. The only study intervention for all children in the hospital cohort was a daily dose of 100 mg thiamine (CBF Pharmaceutical Factory) by intramuscular or intravenous administration for a minimum of 3 d or longer if necessary as judged by the hospital physicians. TRD was determined based on recovery from TDD-compatible symptoms (as distinct from recovery that would be expected from a separate concurrent illness) within 4–12 h after administration of thiamine, as judged by 3 expert pediatricians. These experts independently reviewed the case reports of each hospitalized child to judge whether a child had TRD using a 4-point scale (classic beriberi, probable TRD, possible TRD, and not likely TRD). The categories classic beriberi and probable TRD were subsequently combined as “TRD,” and possible TRD and not likely TRD were combined as “non-TRD.” Details of this categorization process have been presented elsewhere [20,26].

A similar physical assessment was performed at one-time point among children in the community cohort. Information on socioeconomic characteristics, birth and health history, and dietary and health practices was collected for all child-mother pairs in the hospital cohort and the community comparison group [26,28,29]. Anthropometric assessments were completed following recommended protocols in both cohorts [30]. Among children, stunting, underweight, and wasting were defined as length-for-age *z*-scores, weight-for-age *z*-scores, and weight-for-length *z*-scores < −2 SD [31].

### Blood collection and laboratory analyses

Prior to the first thiamine administration, 5 mL venous blood was collected from children into 6-mL BD EDTA evacuated tubes (ref 367863; Becton, Dickinson & Company), and 10 mL venous blood was collected from the women into 10-mL BD EDTA evacuated tubes (ref 368589; Becton, Dickinson & Company). Blood samples were processed in the hospital laboratory or a temporary mobile laboratory in the community to ensure that erythrocytes were washed within 2 h of the blood draw. For later assessment of ThDP, whole blood was aliquoted into amber microcentrifuge tubes and stored at −80°C. The remaining blood was centrifuged at ∼1200 × *g* for 10 min (DM0412S, DLAB Scientific Co., Ltd.). Plasma was aliquoted, and after removing the buffy coat, packed erythrocytes were washed 3 times in 0.9% saline and centrifuged at ∼1900 × *g* for 10 min each time. All samples aliquoted in the community were frozen in an electronic cool box at −20°C and transferred to the −80°C freezer upon arrival at the hospital.

Complete blood count was determined using a hematology analyzer (BC-3000 Plus Auto Hematology Analyzer, Shenzhen Mindray Bio-Medical Electronics Co. Ltd.). Due to technical problems encountered with the community samples, complete blood count results are only available from the hospital cohort. The concentration of ThDP was determined using the thiochrome reaction coupled with the high-performance liquid chromatography fluorescence detection (HPLC-FLD) method [32], and the assay for ETK activity was completed using an UV spectrophotometer at the Nutritional Biomarker Laboratory at the University of Cambridge (United Kingdom). The detailed ETK assay protocol was recently published [23,33]. Briefly, the activity of transketolase was measured in several steps: *1*) basal ETK activity was measured in erythrocytes; *2*) ETK activity was measured following addition of excess exogenous ThDP (stimulated ETK activity); and *3*) ETKac was calculated by dividing the stimulated ETK activity by the basal ETK activity. Erythrocyte glutathione reductase activity coefficient (EGRac), a functional indicator of riboflavin status, was measured in erythrocytes before and after incubation with added flavin adenine dinucleotide, also at the Nutritional Biomarker Laboratory [34]. Plasma C-reactive protein, α-1-acid-glycoprotein, ferritin, and soluble transferrin receptor (sTfR) were analyzed by ELISA at the VitMin Laboratory [35].

Analytical imprecision for ThDP was assessed using 2 single-donor, whole blood quality control (QC) samples with mean measured concentrations of 167 and 83 nmol/L, and a third in-house QC material prepared by dilution of whole blood with phosphate-buffered saline, which had a concentration of 55 nmol/L. Between-run percent coefficient of variation (%CV) was ≤7%. In addition, the whole blood calibration standard for vitamin B_1_ (Chromsystems) was measured and had a between-run %CV of 8%. For both ETKac and EGRac, 3 QC samples, prepared from single-donor samples and stored at −70°C as erythrocyte lysates, were measured with each batch. Due to the difficulty of obtaining blood samples in sufficient quantity from people with high ETKac, all QCs had ETKac values <1.2; between-run %CVs were ≤3%.

### Human milk collection and laboratory analyses

Because mothers of breastfed children with suspected beriberi are routinely prescribed oral thiamine supplementation following recommendations by the World Health Organization [36], we collected milk and maternal blood prior to any thiamine supplementation provided by the hospital. After cleaning the breast with a sanitary wipe (Hygea Obstetrical Towelette, PDI Healthcare), milk from the fuller breast was collected into a human milk bag with an electronic hospital-grade breast pump (Symphony pump, Medela AG) until the breast was emptied. Because circadian differences in concentrations of thiamine are small [37], the milk sample was collected at any time of day. Time of day, time since last meal, breast side, time since last emptying of that side, and milk volume were recorded. Samples were mixed well before aliquoting into 1.5-mL amber microcentrifuge tubes and frozen at −80°C until analysis at the USDA, ARS Western Human Nutrition Research Center (Davis, CA, United States). Samples were analyzed by HPLC-FLD after derivatization of the thiamine vitamers into their trichome esters [38]. An in-house pooled human milk sample was used for QC with each analytical run [39], and the interday (*n* = 56) variations of free thiamine, thiamine monophosphate, and ThDP were 8.1%, 7.9%, and 19.7%. MTh concentrations were calculated as: free thiamine + (thiamine monophosphate × 0.871) + (ThDP × 0.707).

### Primary outcome variable definitions

#### Thiamine diphosphate

ThDP concentration is reported as nmol/L whole blood and normalized for hematocrit (nmol/L red blood cells [RBCs]) or hemoglobin (nmol/g hemoglobin). There is presently no international consensus for a ThDP cutoff to define deficiency. For the purpose of the present study, we defined low ThDP as <95 nmol/L, as previously suggested for adults [40,41].

#### Erythrocyte transketolase

Because diseases and administration of drugs and hormones may affect the apoenzyme levels and confound interpretation of ETKac [42], and previous studies found that basal ETK activity discriminated infantile beriberi cases from controls better than ETKac [43], we explored the use of basal ETK activity, stimulated ETK activity, and ETKac. There is presently no international consensus on a cutoff to define deficiency, but we applied the common thresholds used for ETKac: high risk, >1.25; moderate risk, 1.15 to 1.25; and low risk <1.15 [13,22,24].

#### Human milk thiamine concentration

Based on recent data from the Mothers, Infants, and Lactation Quality (MILQ) study [44], 200 μg/L exceeds the 90^th^ percentile of concentrations in milk from unsupplemented, well-nourished women from 4 countries. Here, we defined low MTh total thiamine concentration as <90 μg/L, which is the 25^th^ percentile of values from the MILQ study (unpublished data, personal communication L.H. Allen, USDA, ARS Western Human Nutrition Research Center, Davis, CA, March 14, 2024).

### Statistical analysis

A statistical analyses plan was developed and published prior to the analyses [45]. All analyses were completed in Stata v16.1 (StataCorp LLC). To ensure we considered baseline thiamine status and did not include participants with biomarker concentrations artificially increased by recent treatment, we excluded thiamine biomarkers for women and children who reported supplementation prior to the blood draw and/or had a free thiamine concentration greater than the 90^th^ percentile of the study sample (>202.7 nmol/L), a conservative cutoff. As a further robustness check, we also conducted sensitivity analyses excluding study participants who reported supplementation prior to the blood draw and/or had free thiamine concentration >80^th^ percentile. Analyses considering MTh were limited to women breastfeeding infants <6 mo of age.

For the present analyses, a complete-case analysis approach was taken using all available data from the Lao Thiamine Study. Although sample size differed by biomarker and physiological group, the available sample size of 287 hospital children with thiamine biomarker data provided 80% power to detect associations with a correlation as small as 0.17.

To compare thiamine status by cohort, we summarized the distribution of each biochemical indicator using median and quartiles separately within the hospitalized cohort with TRD, the hospitalized cohort without TRD, and the community control group. Concentrations of each biomarker were then compared among the cohorts using an analysis of covariance model controlling for child age and sex. Variables were log transformed prior to analysis. The prevalence of the binary categorization of each biomarker was compared across cohorts using a logistic regression model controlling for child age and sex. To develop an exploratory cutoff associated with TRD, we used an area under the receiver operating characteristic curve (AUROC) framework with optimal cutoffs selected via the closest-to-(0,1) corner cutpoint approach [46]. Spearman correlations were estimated for each pairwise combination of thiamine biomarkers to assess the strength of their associations. The same AUROC framework approach was used to explore ThDP cutoffs corresponding to elevated ETKac status, both in the full sample of women and children as well as those stratified by cohort.

## Results

### Characteristics of children and their mothers

**FIGURE 1:**
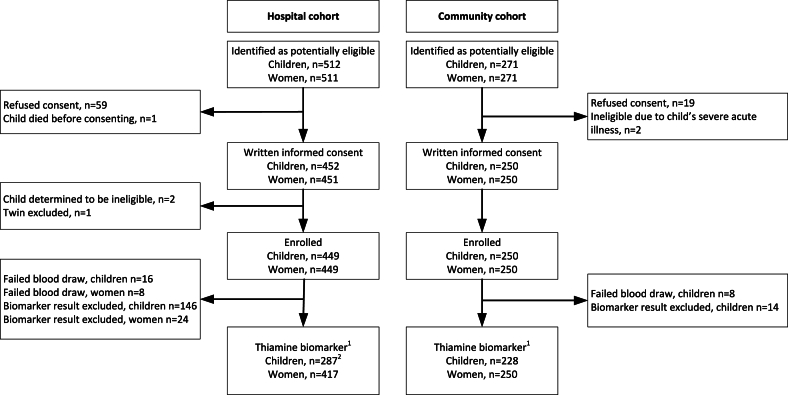
Flow chart of study participants. TRD, thiamine responsive disorder. ^1^Number of study participants with ≥1 thiamine biomarker result and/or human milk thiamine concentration. ^2^Of 287 hospitalized children, 12 could not be included in objective 1 as they did not have a TRD status assigned: 6 died and 2 withdrew consent during the hospital stay; 1 did not have required follow-up assessments; and for 3 of these children, the pediatricians did not reach consensus TRD status. All data was included in objectives 2 and 3.

In the hospital cohort, 512 children were identified as potentially eligible, and 449 child-mother dyads were enrolled (Figure 1). However, 146 hospitalized children and 24 of their mothers had to be excluded from the present analysis, of which 99% had free thiamine concentrations above the 90^th^ percentile and/or because they had reportedly received thiamine prior to their blood draws (Supplemental Table S2). Overall, 287 children and 417 mothers in the hospital cohort had ≥1 thiamine biomarker and/or MTh result. Of 287 hospitalized children, 12 could not be included in objective 1 as they did not have a TRD status assigned: 6 died during the hospital stay, 2 withdrew consent, 1 did not have required follow-up assessments, and for 3 of these children, the pediatricians did not reach consensus TRD status. In the community comparison group, 271 child-mother dyads were considered potentially eligible, 250 were enrolled, and 228 children and 250 women had ≥1 thiamine biomarker and/or MTh result.

Among dyads in which either the mother and/or child had ≥1 thiamine biomarker, children in the hospital cohort were 4.3 ± 3.5 (mean ± SD) mo of age (Table 1 [28,29,47]). In the frequency-matched community comparison group, mean age was 4.5 ± 3.2 mo. Mothers were 24.8 ± 6.7 y old in the hospital and 24.8 ± 6.2 y in the community. There were significant differences in the breastfeeding practices between the hospital and the community (*P* < 0.001), but the percentage of exclusively breastfed infants <6 mo of age was comparable (77.8% in the hospital and 78.9% in the community). Only 14.7% of women achieved minimum dietary diversity for women, and among children 6 to 18 mo of age, only 14.8% achieved minimum dietary diversity. Dietary diversity was significantly lower among women and children in the hospital cohort. More hospitalized children were stunted than in the community comparison group (32.3% compared with 14.9%, *P* < 0.001), and this was also true for underweight (28.6% and 8.8%, *P* < 0.001) and wasting (11.6% compared with 2.0%, *P* < 0.001). The prevalence of thiamine, riboflavin, and iron deficiencies was high among children and their mothers (Table 2 [48,49]).

**TABLE 1:** Baseline characteristics of infants and young children and their mothers in the hospital cohort and community comparison group of the Lao Thiamine Study among participants from which child and/or maternal thiamine biomarkers were available

Characteristics	All	Hospital	Community	*P*
*N* mother-child dyads^1^	688	438	250	
Maternal characteristics				
Age, y	24.8 (6.5)	24.8 (6.7)	24.8 (6.2)	0.971
Biological mother, *n* (%)	684 (98.7)	434 (98.0)	250 (100.0)	0.161
Pregnant, *n* (%)	11 (1.6)	7 (1.6)	4 (1.6)	0.999
Province of residence, *n* (%)				0.237
Luang Prabang	627 (91.1)	400 (91.3)	227 (90.8)	
Oudomxay	39 (5.7)	21 (4.8)	18 (7.2)	
Xayaboury	13 (1.9)	8 (1.8)	5 (2.0)	
Other	9 (1.3)	9 (2.1)	0 (0.0)	
Ethnic group, *n* (%)				<0.001
Lao	103 (15.7)	48 (11.4)	55 (23.3)	
Khmu	224 (34.1)	103 (24.5)	121 (51.3)	
Hmong	329 (50.2)	269 (64.0)	60 (25.4)	
Education, *n* (%)				<0.001
No formal education	132 (19.4)	102 (23.7)	30 (12.0)	
Some/completed primary	211 (31.0)	124 (28.8)	87 (34.8)	
Some/completed secondary	292 (42.9)	184 (42.8)	108 (43.2)	
College/university	45 (6.6)	20 (4.7)	25 (10.0)	
Occupation, *n* (%)				<0.001
Does not work	9 (1.3)	4 (0.9)	5 (2.0)	
Housewife	140 (20.6)	73 (17.0)	67 (26.8)	
Farmer	432 (63.5)	308 (71.6)	124 (49.6)	
Unskilled laborer	3 (0.4)	2 (0.5)	1 (0.4)	
Skilled worker^2^	94 (13.8)	42 (9.8)	52 (20.8)	
Household SES index^3^	0.0 (1.0)	−0.1 (1.0)	0.2 (1.0)	<0.001
Food insecurity category, *n* (%)				<0.001
None to mild	451 (66.3)	260 (60.5)	191 (76.4)	
Moderate to severe	229 (33.7)	170 (39.5)	59 (23.6)	
Gravidity, *n* (%)	2.6 (1.8)	2.7 (2.0)	2.3 (1.5)	0.005
ANC visits, *n* (%)				<0.001
0–3	222 (33.2)	174 (41.4)	48 (19.4)	
4–7	282 (42.2)	169 (40.2)	113 (45.6)	
≥8	164 (24.6)	77 (18.3)	87 (35.1)	
Reported prenatal supplementation, *n* (%)	571 (84.0)	343 (79.8)	228 (91.2)	<0.001
Dietary diversity score	3.2 (1.4)	3.0 (1.4)	3.5 (1.3)	<0.001
Achieved MDD-W^4^, *n* (%)	100 (14.7)	54 (12.5)	46 (18.4)	0.037
Restricted diet postpartum, *n* (%)				0.876
Never restricted	55 (8.0)	36 (8.2)	19 (7.6)	
Previously restricted	346 (50.4)	184 (42.1)	102 (40.8)	
Presently restricting	286 (41.6)	217 (49.7)	129 (51.6)	
Weight, kg	48.9 (7.1)	48.2 (6.9)	50.1 (7.4)	<0.001
Height, cm	149.5 (5.6)	148.6 (5.5)	151.0 (5.5)	<0.001
BMI, kg/m^2^	21.9 (2.8)	21.8 (2.7)	22.0 (2.9)	0.471
BMI <18.5 kg/m^2^, *n* (%)	55 (8.3)	36 (8.7)	19 (7.7)	0.647
MUAC, cm	24.3 (2.5)	24.2 (2.6)	24.4 (2.4)	0.240
Child characteristics				
Age, mo	4.4 (3.4)	4.3 (3.5)	4.5 (3.2)	0.560
Male, *n* (%)	408 (59.4)	264 (60.3)	144 (57.8)	0.531
Breastfeeding status among <6 mo, *n* (%)				<0.001
Exclusive breastfeeding	406 (78.2)	260 (77.8)	146 (78.9)	
Predominant breastfeeding^5^	31 (6.0)	25 (7.5)	6 (3.2)	
Mixed milk feeding^6^	41 (7.9)	30 (9.0)	11 (5.9)	
Continued breastfeeding^7^	24 (4.6)	4 (1.2)	20 (10.8)	
No longer breastfeeding	17 (3.3)	15 (4.5)	2 (1.1)	
Breastfeeding status among 6–18 mo, *n* (%)				0.011
Exclusive breastfeeding	13 (7.7)	10 (9.6)	3 (4.6)	
Predominant breastfeeding^5^	14 (8.3)	12 (11.5)	2 (3.1)	
Mixed milk feeding^6^	2 (1.2)	2 (1.9)	0 (0.0)	
Continued breastfeeding^7^	116 (68.6)	61 (58.7)	55 (84.6)	
No longer breastfeeding	24 (14.2)	19 (18.3)	5 (7.7)	
Complementary feeding indicators^8^, *n* (%)				
Minimum dietary diversity^9^	25 (14.8)	6 (5.8)	19 (29.2)	<0.001
Minimum meal frequency	81 (57.0)	32 (39.0)	49 (81.7)	<0.001
Minimum acceptable diet	18 (12.7)	1 (1.2)	17 (28.3)	<0.001
Length, cm	59.7 (6.5)	59.1 (6.7)	60.6 (6.0)	0.003
Weight, kg	5.7 (1.5)	5.5 (1.5)	6.0 (1.5)	<0.001
MUAC, cm	12.8 (1.4)	12.4 (1.5)	13.3 (1.1)	<0.001
Head circumference, cm	39.7 (3.1)	39.4 (3.1)	40.2 (3.0)	0.001
Length-for-age *z*-score	−1.3 (1.4)	−1.5 (1.5)	−1.0 (1.0)	<0.001
Weight-for-age *z*-score	−1.1 (1.3)	−1.4 (1.5)	−0.7 (0.9)	<0.001
Weight-for-length *z*-score	−0.2 (1.2)	−0.3 (1.4)	0.0 (1.0)	0.002
Stunted, *n* (%)	168 (25.6)	131 (32.3)	37 (14.9)	<0.001
Wasted, *n* (%)	52 (8.0)	47 (11.6)	5 (2.0)	<0.001
Underweight, *n* (%)	138 (21.1)	116 (28.6)	22 (8.8)	<0.001

Abbreviations: ANC, antenatal care; BMI, body mass index; MDD-W, minimum dietary diversity for women; MUAC, mid-upper arm circumference; SES, socioeconomic status.

1 Data shown if either mother or child has thiamine biomarker and/or human milk thiamine concentration. Sample size for different outcomes may vary. Values are mean (SD) unless otherwise indicated.

2 Merchant, business, or government employee.

3 Self-reported indicators of SES included education and occupation of the mother and household head, household size and composition, housing characteristics, access to utilities, and household ownership of assets and land. These proxy indicators were used to estimate household SES index using principal component analysis.

4 Consumption of ≥5 of 10 defined food groups in the previous 24 h before going to the hospital was considered as meeting the MDD-W [47].

5 Breastfeeding with certain liquids (water, water-based drinks, fruit juice) [28].

6 Breastfeeding with formula and/or animal milk [29].

7 Breastfeeding with soft foods or other combinations [29].

8 Complementary feeding indicators [29] among 6–18 mo old study participants.

9 Consumption of ≥5 out of 8 defined food groups in the previous 24 h before going to hospital was considered as meeting the minimum dietary diversity [29].

**TABLE 2:** Thiamine and other micronutrient status biomarkers among children and their mothers in the hospital cohort and community comparison group of the Lao Thiamine Study

Characteristics	Children			Women		*P*
	Hospital	Community	*P*	Hospital	Community	
*N*^1^	287	228		417	250	
Thiamine status						
Whole blood ThDP^2^, nmol/L	66.9 (41.4, 96.9)	64.1 (50.0, 85.3)	0.012	70.2 (55.1, 70.2)	73.6 (54.6, 94.6)	0.402
Low ThDP <95 nmol/L, *n* (%)	211 (73.8)	185 (81.9)	0.030	343 (82.5)	190 (76.0)	0.044
Whole blood ThDP/hematocrit^3^^,^^4^,nmol/L RBC	2.22 (1.34, 3.21)	—	—	1.90 (1.49, 2.36)	—	—
Whole blood ThDP/hemoglobin^3^^,^^4^,nmol/g hemoglobin	6.37 (3.89, 9.37)	—	—	5.62 (4.41, 7.07)	—	—
ETKac	1.25 (1.11, 1.48)	1.22 (1.12, 1.37)	<0.001	1.29 (1.18, 1.44)	1.23 (1.15, 1.30)	<0.001
Elevated ETKac >1.25, *n* (%)	122 (49.8)	84 (45.4)	0.367	249 (59.7)	105 (42.0)	<0.001
Human milk total thiamine^5^, μg/L	—	—	—	82.1 (43.2, 135.5)	114.6 (80.1, 150.6)	0.013
Human milk totalthiamine^5^ <90 μg/L, *n* (%)	—	—	—	159 (54.1)	57 (31.3)	<0.001
Riboflavin status						
EGRac	1.48 (1.26, 1.95)	1.51 (1.35, 1.73)	0.992	2.31 (1.88, 2.85)	2.12 (1.70, 2.63)	<0.001
Elevated EGRac >1.3, n (%)	167 (68.4)	147 (79.5)	0.011	405 (97.1)	235 (94.0)	0.046
Iron status						
Ferritin, unadjusted, μg/L	141.6 (61.9, 212.4)	86.9 (29.8, 172.3)	<0.001	48.5 (15.3, 87.4)	56.4 (27.3, 96.0)	0.231
Ferritin, corrected^6^, μg/L	—	—	—	29.6 (9.9, 53.8)	33.3 (17.5, 59.8)	0.051
Low ferritin^6^^,^^7^, *n* (%)	19 (7.0)	18 (8.5)	0.529	133 (31.9)	51 (20.4)	0.001
sTfR, mg/L	6.0 (4.1, 8.5)	5.4 (3.9, 7.3)	0.001	5.5 (4.4, 8.4)	4.8 (4.0, 6.4)	<0.001
Elevated sTfR >8.3 mg/L, *n* (%)	71 (26.0)	33 (15.6)	0.006	105 (25.2)	36 (14.4)	0.001
Inflammation status						
CRP, mg/L	5.9 (1.2, 24.8)	0.4 (0.1, 1.9)	<0.001	0.8 (0.3, 2.0)	0.7 (0.3, 1.7)	0.136
Elevated CRP >5 mg/L, *n* (%)	147 (53.8)	24 (11.3)	<0.001	50 (12.0)	20 (8.0)	0.104
AGP, g/L	0.9 (0.7, 1.4)	0.5 (0.4, 0.7)	<0.001	0.7 (0.6, 0.8)	0.6 (0.5, 0.8)	<0.001
Elevated AGP >1 g/L, *n* (%)	120 (44.0)	13 (6.1)	<0.001	55 (13.2)	10 (4.0)	<0.001
Anemia status						
Hemoglobin^4^, g/L	107.4 (19.0)	—	—	125.2 (19.1)	—	—
Anemia^4^^,^^8^, *n* (%)	150 (53.4)	—	—	130 (31.6)	—	—

Abbreviations: AGP, α-1-acid glycoprotein; CRP, C-reactive protein; EGRac, erythrocyte glutathione reductase activity coefficient; ETKac, erythrocyte transketolase activity coefficient; IQR, interquartile range; RBC, red blood cell; SD, standard deviation; sTfR, soluble transferrin receptor; ThDP, thiamine diphosphate.

Results shown as *n* (%), mean (SD), and median (IQR).

1 Results shown for children or women who have thiamine biomarker results. Sample size varies by outcome.

2 For conversion of whole blood ThDP to μg/L, multiply nmol/L × 0.42531.

3 Whole blood ThDP normalized for hematocrit (nmol/L RBC) and hemoglobin (nmol/g hemoglobin). Normalized results were strongly correlated with the raw values (correlation >0.9).

4 No hematocrit or hemoglobin results available for community comparison group because of technical errors with complete blood count analysis.

5 Human milk thiamine among mothers of infants <6 mo of age.

6 Ferritin concentration for women corrected for inflammation following recommendations by Biomarkers Reflecting Inflammation and Nutritional Determinants of Anemia (BRINDA) [48]. As per BRINDA guidance, adjustments for children were not indicated as associations were in the opposite direction of biologically expected inflammation effects.

7 Low ferritin (<15 μg/L) for women and (<12 μg/L) for children [49].

8 Hemoglobin cutoffs: <120 g/L for non-pregnant women; <110 g/L for pregnant women and children.

### TRD and thiamine status

Among the hospitalized children included in the present study, 18.2% children were classified as classic beriberi, 37.5% as probable TRD, 37.1% as possible TRD, and 7.3% as not likely TRD, resulting in 55.6% of children being classified as TRD and 44.4% as non-TRD. This TRD prevalence differs from the TRD prevalence of 60.8% for the complete hospital cohort reported elsewhere [20] because more severely ill children had to be excluded from the present analysis due to urgent thiamine administration prior to the blood draw (Supplemental Table S2). As we reported previously in more detail [20], among hospitalized children, signs and symptoms consistent with cardiac beriberi were most common, and relatively few children presented with signs and symptoms consistent with neurological TDD.

The prevalence of ThDP <95 nmol/L was 77.3% and elevated ETKac (>1.25) was 47.9% among all children combined, and 45.4% of mothers had low MTh (<90 μg/L) (Table 2). Whole blood ThDP normalized for hematocrit (nmol/L RBC) and hemoglobin (nmol/g hemoglobin) was strongly correlated with the raw values (correlation > 0.9). Median (IQR) ThDP was significantly lower among TRD cases (64.2 nmol/L; IQR: 40.0, 89.9 nmol/L) than non-TRD cases in the hospital cohort (72.2 nmol/L; IQR: 50.3, 116.1 nmol/L; *P* = 0.001; Table 3). However, ThDP concentration was not significantly different between TRD cases and the community comparison group (64.1 nmol/L; IQR: 50.0, 85.3 nmol/L; *P* = 0.225). Median (IQR) ETKac among TRD cases was significantly higher (1.27 nmol/L; IQR: 1.14, 1.59 nmol/L) than among non-TRD (1.22 nmol/L; IQR: 1.08, 1.44 nmol/L; *P* = 0.005) and the frequency-matched community group (1.22 nmol/L; IQR: 1.12, 1.37 nmol/L; *P* < 0.001). Basal ETK activity was significantly different between TRD and non-TRD cases, but neither one of these was significantly different from community children, and there were no overall significant differences in stimulated ETK activity (Table 3). There was also substantial overlap in ThDP concentrations, ETKac, and basal ETK activity when we explored the more detailed clinical classifications of classic beriberi, probable, possible, and not likely TRD (Supplemental Figure S1). Among mothers of infants <6 mo of age, median (IQR) MTh was 75.0 μg/L (IQR: 38.3, 134.0 μg/L) among hospital mothers of TRD cases, 94.5 μg/L (IQR: 51.3, 140.9 μg/L) among hospital mothers of non-TRD cases, and 114.6 μg/L (IQR: 80.1, 150.6 μg/L) among the community mothers. Although MTh was not significantly different between mothers of TRD and non-TRD cases (*P* = 0.151), MTh among community mothers was significantly higher than among mothers of both TRD (*P* < 0.001) and non-TRD cases (*P* = 0.003). Almost half of all women had MTh concentrations <90 μg/L, although the prevalence differed significantly by TRD status and cohort (31.3%–57.1%, *P* < 0.001).

**TABLE 3:** Median concentration of thiamine biomarkers and low thiamine status among children, by clinically defined TRD status in the hospital cohort and a frequency-matched community cohort, and thiamine human milk concentration among breastfeeding mothers of infants <6 months of age^1^

Biomarker	Median (IQR)			*P*
	TRD	Non-TRD	Community	Overall
*N*	152	122	226	
Whole blood ThDP, nmol/L	64.2 (40.0, 89.9)^a^	72.2 (50.3, 116.1)^b^	64.1 (50.0, 85.3)^a^	0.004
Low ThDP <95 nmol/L, n (%)	119 (78.3)^a^	84 (68.9)^b^	185 (81.9)^a^	0.014
*N*	133	101	185	
Basal ETK activity, U/g Hb/min	0.40 (0.26, 0.55)^a^	0.44 (0.30, 0.62)^b^	0.40 (0.32, 0.53)^ab^	0.036
Stimulated ETK activity, U/g Hb/min	0.52 (0.39, 0.65)^ab^	0.52 (0.41, 0.74)^a^	0.51 (0.42, 0.61)^b^	0.067
ETKac	1.27 (1.14, 1.59)^a^	1.22 (1.08, 1.44)^b^	1.22 (1.12, 1.37)^b^	<0.001
Elevated ETKac >1.25, n (%)	72 (53.3)	45 (44.6)	84 (45.4)	0.312
*N*	196	82	182	
Human milk total thiamine^2^, μg/L	75.0 (38.3, 134.0)^a^	94.5 (51.3, 140.9)^a^	114.6 (80.1, 150.6)^b^	<0.001
Human milk total thiamine^2^, <90 μg/L, *n* (%)	112 (57.1)^a^	39 (47.6)^a^	57 (31.3)^b^	<0.001

Abbreviations: ANCOVA, analysis of covariance; ETK, erythrocyte transketolase; ETKac, erythrocyte transketolase activity coefficient; Hb, hemoglobin; IQR, interquartile range; ThDP, thiamine diphosphate; TRD, thiamine responsive disorder.

Results shown as *n* (%) and median (IQR).

1 Biomarker concentrations were log transformed for analysis. The ANCOVA model was controlled for child age and sex. Superscript lowercase letters indicate statistically significant pairwise differences (*P* < 0.05).

2 Human milk thiamine among mothers of infants <6 mo of age.

### Exploring thiamine status cutoffs associated with TRD status

Due to poor sensitivity and specificity ranging from 0.53 to 0.59, efforts to explore potential cutoffs for increased risk of TRD using the AUROC framework resulted in uninformative cutoffs for whole blood ThDP, basal ETK activity and stimulated ETK activity, ETKac, and MTh (Table 4). In other words, none of the explored thiamine biomarkers were able to distinguish TRD status reliably, suggesting TRD status is driven by more factors than biomarker concentration.

**TABLE 4:** AUROC, potential cutoff, and associated sensitivity and specificity to predict TRD status and elevated ETKac

Outcome	Predictor	Subsample	N	AUROC (95% CI)	Optimal cutoff	Sensitivityat cutoff	Specificityat cutoff
TRD status	ThDP	Hospitalized children	274	0.59 (0.52, 0.67)	60.0 nmol/L	0.47	0.65
TRD status	Basal ETK activity	Hospitalized children	234	0.56 (0.48, 0.63)	0.42 U/g Hb/min	0.60	0.53
TRD status	Stimulated ETK activity	Hospitalized children	234	0.53 (0.45, 0.60)	0.60 U/g Hb/min	0.66	0.41
TRD status	ETKac	Hospitalized children	234	0.57 (0.50, 0.64)	1.25	0.56	0.55
TRD status	MTh	Hospitalized children	278	0.55 (0.48, 0.62)	86.1 μg/L	0.56	0.59
Elevated ETKac	ThDP	All children	427	0.86 (0.83, 0.90)	64.1 nmol/L	0.79	0.80
Elevated ETKac	ThDP	Hospitalized children	244	0.90 (0.86, 0.94)	68.2 nmol/L	0.83	0.82
Elevated ETKac	ThDP	Community children	183	0.81 (0.75, 0.87)	65.5 nmol/L	0.83	0.72
Elevated ETKac	ThDP	All women	666	0.66 (0.62, 0.71)	74.4 nmol/L	0.68	0.61
Elevated ETKac	ThDP	Hospital women^1^	416	0.73 (0.68, 0.78)	72.2 nmol/L	0.71	0.72
Elevated ETKac	ThDP	Community women	250	0.56 (0.49, 0.63)	85.0 nmol/L	0.75	0.43

Abbreviations: AUROC, area under the receiver operating characteristic curve; CI, confidence interval; ETK, erythrocyte transketolase; ETKac, erythrocyte transketolase activity coefficient; Hb, hemoglobin; MTh, human milk thiamine; ThDP, whole blood thiamine diphosphate; TRD, thiamine responsive disorder.

1 Mothers of hospitalized children.

### Exploring whole blood ThDP concentrations associated with ETKac >1.25

Whole blood ThDP and ETKac were well correlated in hospital (*r* = −0.84) and community (*r* = −0.64) children and less strongly among the mothers in the hospital (*r* = −0.51) and community (*r* = −0.19) cohorts (Supplemental Figure S2). Among all children combined, ThDP was strongly predictive of an ETKac cutoff of >1.25 with an AUROC of 0.86 (95% confidence interval [CI]: 0.83, 0.90); a ThDP cutoff of 64.1 nmol/L had a sensitivity of 0.79 and specificity of 0.80 (Table 4). Similarly, among mothers of the hospital cohort, ThDP was strongly predictive of an ETKac cutoff of >1.25 with an AUROC of 0.73 (95% CI: 0.68, 0.78); a ThDP cutoff of 72.2 nmol/L had a sensitivity of 0.71 and specificity of 0.72. In contrast, among women in the community, AUROC was 0.56 (95% CI: 0.49, 0.63) with a proposed ThDP cutoff of 85 nmol/L but a sensitivity of 0.75 and a specificity 0.43.

All of the above reported results were robust when restricting the sample to study participants with free thiamine concentration >80^th^ percentile.

## Discussion

In the present cohort study among infants and young children hospitalized for cardiovascular, respiratory, neurological, and/or behavioral disorders suggestive of thiamine deficiency and a frequency-matched, community comparison group, the prevalence of low ThDP, elevated ETKac, and low MTh was high among the children as well as their mothers, regardless of study cohort, suggesting that thiamine deficiency is a severe public health concern in the broader community. More than half of the hospitalized children were classified as TRD [20]; although the median ThDP was significantly lower among TRD cases than non-TRD cases in the hospital cohort, ThDP concentration was not significantly different between TRD cases and the community comparison group. In contrast, the median ETKac among TRD cases was significantly higher than that among non-TRD cases and the frequency-matched community group. Among mothers of infants <6 mo of age, the median MTh was significantly higher in the community than among mothers in the hospital cohort of both TRD and non-TRD cases. However, because of substantial overlap and small differences of all these biomarker concentrations and ratios among TRD and non-TRD cases, efforts to set a cutoff to predict TRD status failed due to poor sensitivity and specificity. Thus, these biomarkers have limited usefulness for identifying clinical manifestations of thiamine deficiency in a setting with a high prevalence of low thiamine status, and other biomarkers such as lactate concentration and/or metabolomics should be explored further.

The large overlapping range in all thiamine biomarkers may be due to the fact that thiamine deficiency can be caused by inadequate thiamine intake [4] as well as increased cellular demand for thiamine during critical illnesses such as severe infections [14]. During illness, the metabolic requirement increases, and children need significantly more thiamine to provide enough cofactor for complete oxidation of glucose. Because each acute illness varies in severity, and the inflammatory cascade varies in each individual, large variability in thiamine requirement and status may be expected. This may be further complicated by genetic variability and the complex interplay between gut microbiota and human thiamine metabolism [50].

Our finding that ThDP was not clinically useful in identifying children likely to respond favorably to thiamine administration confirms the findings from 3 previous studies in which mean ThDP concentrations among hospitalized Cambodian infants diagnosed with beriberi, tachypnea, or cardiac dysfunction, respectively, were not significantly different from their matched hospital controls [17,18,51]. In contrast, 2 small studies in Kashmir found a significant difference in whole blood ThDP between thiamine responders and nonresponders among adults presenting with either neuropathy suggestive of TDD or high-output heart failure [52,53].

Even though there were statistically significant differences in median ETKac and basal ETK activity between TRD and non-TRD cases, neither ETKac nor basal ETK activity were numerically different enough to be clinically useful to identify individual children with TRD due to the wide range of ETKac ratios and basal ETK activity between the different cohorts. Thus, attempts to define an ETKac and basal ETK activity cutoff associated with TRD in the present study failed. Similarly, among infants with beriberi and age-matched afebrile and febrile controls in Vientiane, the capital of Lao PDR, ETKac did not discriminate between cases and controls [43]. However, basal ETK activity ≤0.59 μmol/min/g hemoglobin was proposed as predicting infantile beriberi [43]. We could not confirm the clinical usefulness of basal ETK activity because basal ETK activity in the community comparison group was not significantly different from that in the TRD cases. Besides the inclusion of a community comparison group, an additional major difference between the present study and the study in Vientiane was that the infants in Vientiane were diagnosed with beriberi, the most severe clinical manifestations of thiamine deficiency, whereas in the present study, children were diagnosed based on a favorable response to therapeutic thiamine administration. Thus, in the present study, a wider range of TDD signs and symptoms as well as the clinical course of the infants after hospitalization were evaluated. Although we were not able to define cutoffs for any of the candidate thiamine biomarkers to identify individual children with TRD, ETKac added value in the prediction model for TRD, as we previously reported [20]. Other predictors of TRD identified were hoarse voice/loss of voice, exclusive or predominant breastfeeding, cyanosis, not making eye contact, and no diarrhea in the previous 2 wk, as well as cranial ultrasound when considering predictors potentially available in more highly resourced settings [20].

Low thiamine concentration in human milk is a well-established risk factor for thiamine deficiency among breastfed infants [9]. Although MTh was significantly different between mothers of infants <6 mo of age in the hospital compared with those in the community, MTh did not significantly differ between mothers of infants with or without TRD. Thus, MTh was not clinically useful to identify children likely to respond positively to therapeutic thiamine administration. We are not aware of previous studies comparing thiamine concentration in human milk among infants with beriberi or TDD. Mean MTh concentration in the present study (110.6 ± 80.4 μg/L) was slightly lower compared with a recent thiamine supplementation study in Cambodia in which women in the placebo group had mean MTh ranging from 118.8 ± 59.8 μg/L at 4 wk postpartum to 152.5 ± 84.8 μg/L at 24 wk postpartum [54]. In the present study, the prevalence of low MTh, low whole blood ThDP, and elevated ETKac was significantly higher among women in the hospital cohort than in the community cohort. Similarly, women in the hospital cohort were more at risk of iron deficiency based on low inflammation-adjusted ferritin and elevated sTfR and riboflavin deficiency based on elevated EGRac. This is likely due to a range of risk factors related to lower socioeconomic status and higher levels of food insecurity in the hospital cohort.

ETKac is considered a functional indicator of thiamine deficiency, and >1.25 is generally accepted as a cutoff for risk of severe thiamine deficiency, whereas a wide range of cutoffs has been used for ThDP concentrations [22]. In the present study, a ThDP cutoff <64 nmol/L was strongly predictive of an ETKac cutoff of >1.25 among all children and <72.2 nmol/L for women in the hospital cohort with acceptable sensitivity and specificity in both population groups. In contrast, among women in the community, the suggested cutoff <85 nmol/L had poor specificity. It has been reported that, besides thiamine deficiency, other factors may influence the ETKac assay [21], but we are uncertain why women in the community would be so different. The community dyads were selected based on frequency-matching of age, sex, and location of residence to the children in the hospital cohort and not due to any maternal characteristics or other selection criteria. It is difficult to compare our findings with other studies due to the diversity of thiamine biomarkers, specimen type, and result reporting. However, in a study among malaria patients in southern Lao PDR, erythrocyte ThDP <275 ng/g hemoglobin predicted ETKac >1.25 with a sensitivity of 68.5% (95% CI: 54.4, 80.5%) and a specificity of 60.8 (95% CI: 53.2, 68.1%) [55]. A study of 63 patients with acute or chronic medical conditions at risk of thiamine deficiency found that ETKac and erythrocyte ThDP categorized these patients similarly as thiamine or not thiamine deficient [21]. As reviewed recently by Whitfield [22], various adequate ranges of ThDP have been proposed based on distributions in apparently healthy populations [32,40,56]. However, because these studies did not include clinical or functional markers of thiamine status, they are not directly comparable to the current findings and may not be useful to define a cutoff for deficiency.

Although the protocol for the assay to determine ETKac and basal ETK activity have recently been published to promote harmonization of ETK as an indicator for thiamine status [23,33], the required washing of erythrocytes is time consuming and can be particularly challenging when working in community settings where field laboratories have to be set up to allow separation and freezing of the RBC samples within a few hours. In comparison, the collection and processing of whole blood samples for ThDP is easier in the field. Thus, for population representative surveys, whole blood ThDP may be more manageable. Additional research is needed to better understand the association between ETKac and ThDP. Further work is also required to investigate the feasibility of using whole blood in the ETKac assay.

Although the laboratory analysis of ThDP, ETKac, and MTh was validated using internal QC protocols by the respective laboratories, external quality assessment is only available for ThDP in whole blood [57]. Thus, the comparability across different laboratories and the actual prevalence of thiamine deficiency is somewhat uncertain. However, blood specimens collected in the present and other recent studies [7,54], including from the United Kingdom National Diet and Nutrition Survey Rolling Program (2014–2016), were analyzed in the same laboratory [23], and among the adult United Kingdom population, only 1% had ETKac >1.25 compared with 53% among women in the present study, confirming that the study population was at high risk of thiamine deficiency, demonstrating the utility of ETKac as a population marker for thiamine status.

Because many infants and children with life-threatening conditions were treated with thiamine prior to or immediately upon arrival at the hospital, a relatively large number of hospitalized children were excluded from the present analyses because they had received thiamine prior to the blood draw and/or had elevated free thiamine concentration likely due to prior treatment (146 hospital children, 24 hospital women, 2 community children). Results were robust and conclusions were similar in sensitivity analyses when study participants with free thiamine concentration >80^th^ percentile were excluded. A result of excluding children who had received thiamine is that the children included in this present study had less severe clinical signs than the overall study population, which may have limited our ability to distinguish potential differences in biomarkers between patients with and without TRD and between TRD cases and the community comparison group. Another limitation was that the TRD diagnosis was subjectively assigned based on known risk factors of thiamine deficiency and favorable response to thiamine administration, and the clinical response to the therapeutic thiamine administration could not be isolated from other medical interventions provided by the hospital physicians in the context of routine critical care. However, TRD status was assigned by pediatricians with extensive expertise in beriberi after consideration of the speed of recovery and normalization of vital signs within 4 to 12 h of thiamine administration, and there was strong reviewer agreement, as previously reported [20]. Overall, this is the largest prospective cohort study with detailed information correlating clinical history and examination, clinical evolution, and thiamine biomarkers among children hospitalized with clinical signs and symptoms suggestive of TDD. Rigorous data collection and the enrollment of children’s mothers gave strength to the study, and the inclusion of a frequency-matched community comparison group provided a broader population-based perspective.

In conclusion, although there were significant differences between TRD and non-TRD cases in whole blood ThDP, basal ETK activity, and ETKac, there was a wide range in these biomarker concentrations and ratios. Thus, none of these biomarkers proved to be clinically useful in identifying individual children who would respond positively to thiamine administration, and additional point-of-care biomarkers should be explored. Research is needed on the distribution of thiamine biomarkers in various contexts with and without beriberi. Specifically, longitudinal studies are needed to determine the role of acute illnesses and the complex interplay of genetic and other individual factors on the occurrence of beriberi in populations with low thiamine status. A cutoff of 64.1 nmol/L whole blood ThDP predicted ETKac >1.25 among children and 72.2 nmol/L women in the hospital cohort, suggesting that whole blood ThDP may be useful to assess population-level thiamine status in surveys, although additional research is needed to understand why this association was weaker among women in the community. Based on all the thiamine biomarkers collected, there was a high risk of thiamine deficiency in the study population, resulting in a high prevalence of TRD in the hospital cohort. Considering the short- and long-term health risks associated with thiamine deficiency, there is an urgent need to prevent thiamine deficiency in young children and their mothers in the study region and other similar regions, and assessment of thiamine biomarkers in representative population samples coupled with case reports of beriberi can be helpful to identify populations at risk of thiamine deficiency.

## Author contributions

The authors’ responsibilities were as follows – SYH, CDA, KHB, LH, IT, PRF: conceived and designed the study protocol of the Lao Thiamine Study; SYH, TJS: developed the data collection questionnaires; SK: translated all data collection questionnaires to Lao language; XT: programmed all data collection questionnaires; TJS, DS, SK, SYH: planned the local study implementation; TJS, DS: supervised the data collection; LH, IT, PRF: reviewed case reports; KSJ, DH, SRM, DAP, AK, LHA: performed laboratory analyses; CDA: performed statistical analyses; SYH: drafted the manuscript: and all authors: read and approved the final manuscript.

## Conflict of interest

KHB, the spouse of SYH, previously worked and currently serves as a consultant for the Bill & Melinda Gates Foundation. All other authors report no conflicts of interest.

## Funding

This work was supported, in part, by the Bill & Melinda Gates Foundation [grant number INV-009736]. Under the grant conditions of the Foundation, a Creative Commons Attribution 4.0 Generic License has already been assigned to the Author Accepted Manuscript version that might arise from this submission. In addition, the United States Agency for International Development provided financial support to assess riboflavin biomarkers for this article through its flagship multisectoral nutrition project, USAID Advancing Nutrition [contract 7200AA18C00070 awarded to JSI Research & Training Institute, Inc.]. This research was also supported by the NIHR Cambridge Biomedical Research Center [NIHR203312]. The views expressed are those of the authors and not necessarily those of the NIHR or the Department of Health and Social Care.

## Data availability

Data described in the manuscript, code book, and analytic code will be made publicly and freely available without restriction at: https://osf.io/jfke3/.

## References

[1] Liu Z., Farkas P., Wang K., Kohli M.O., Fitzpatrick T.B. (2022). B vitamin supply in plants and humans: the importance of vitamer homeostasis. Plant J.

[2] Brown K.H., Moore S.E., Hess S.Y., McDonald C.M., Jones K.S., Meadows S.R. (2021). Increasing the availability and utilization of reliable data on population micronutrient (MN) status globally: the MN Data Generation Initiative. Am. J. Clin. Nutr..

[3] Whitfield K.C., Smith G., Chamnan C., Karakochuk C.D., Sophonneary P., Kuong K. (2017). High prevalence of thiamine (vitamin B1) deficiency in early childhood among a nationally representative sample of Cambodian women of childbearing age and their children. PLoS Negl. Trop. Dis..

[4] Whitfield K.C., Bourassa M.W., Adamolekun B., Bergeron G., Bettendorff L., Brown K.H. (2018). Thiamine deficiency disorders: diagnosis, prevalence, and a roadmap for global control programs. Ann. N. Y. Acad. Sci..

[5] Johnson C.R., Fischer P.R., Thacher T.D., Topazian M.D., Bourassa M.W., Combs G.F. (2019). Thiamin deficiency in low- and middle-income countries: disorders, prevalences, previous interventions and current recommendations. Nutr. Health.

[6] Smith T.J., Hess S.Y. (2021). Infantile thiamine deficiency in South and Southeast Asia: an age-old problem needing new solutions. Nutr. Bull..

[7] Bourassa M.W., Gomes F., Jones K.S., Koulman A., Prentice A.M., Cerami C. (2022). Thiamine deficiency in Gambian women of reproductive age. Ann. N. Y. Acad. Sci..

[8] Ratsavong K., van Elsacker T., Doungvichit D., Siengsounthone L., Kounnavong S., Essink D. (2020). Are dietary intake and nutritional status influenced by gender? The pattern of dietary intake in Lao PDR: a developing country. Nutr. J..

[9] World Health Organization (1999).

[10] Smith T.J., Tan X., Arnold C.D., Sitthideth D., Kounnavong S., Hess S.Y. (2022). Traditional prenatal and postpartum food restrictions among women in northern Lao PDR, Matern. Child Nutr..

[11] Köhler R., Lambert C., Biesalski H.K. (2019). Animal-based food taboos during pregnancy and the postpartum period of Southeast Asian women - a review of literature. Food Res. Int..

[12] Köhler R., Sae-tan S., Lambert C., Biesalski H.K. (2018). Plant-based food taboos in pregnancy and the postpartum period in Southeast Asia – a systematic review of literature. Nutr. Food Sci.

[13] United States Institute of Medicine (1998).

[14] Hiffler L., Rakotoambinina B., Lafferty N., Martinez Garcia D. (2016). Thiamine deficiency in tropical pediatrics: new insights into a neglected but vital metabolic challenge. Front. Nutr..

[15] Allen L.H. (2012). B vitamins in breast milk: relative importance of maternal status and intake, and effects on infant status and function. Adv. Nutr..

[16] Smith T.J., Johnson C.R., Koshy R., Hess S.Y., Qureshi U.A., Mynak M.L. (2021). Thiamine deficiency disorders: a clinical perspective. Ann. N. Y. Acad. Sci..

[17] Coats D., Shelton-Dodge K., Ou K., Khun V., Seab S., Sok K. (2012). Thiamine deficiency in Cambodian infants with and without beriberi. J. Pediatr..

[18] Keating E.M., Nget P., Kea S., Kuong S., Daly L., Phearom S. (2015). Thiamine deficiency in tachypnoeic Cambodian infants. Paediatr. Int. Child Health.

[19] Lima L.F., Leite H.P., Taddei J.A. (2011). Low blood thiamine concentrations in children upon admission to the intensive care unit: risk factors and prognostic significance. Am. J. Clin. Nutr..

[20] Smith T.J., Arnold C.D., Fischer P.R., Trehan I., Hiffler L., Sitthideth D. (2024). A predictive model for thiamine responsive disorders among infants and young children: results from a prospective cohort study in Lao People's Democratic Republic. J. Pediatr..

[21] Talwar D., Davidson H., Cooney J., St JO’Reilly D. (2000). Vitamin B(1) status assessed by direct measurement of thiamin pyrophosphate in erythrocytes or whole blood by HPLC: comparison with erythrocyte transketolase activation assay. Clin. Chem..

[22] Whitfield K.C., Gibson R.S. (2021). Principles of nutritional assessment.

[23] Jones K.S., Parkington D.A., Cox L.J., Koulman A. (2021). Erythrocyte transketolase activity coefficient (ETKAC) assay protocol for the assessment of thiamine status. Ann. N. Y. Acad. Sci..

[24] Turck D., Bresson J., Burlingame B., Dean T., Fairweather-Tait S.J., EFSA Panel on Dietetic Products Nutrition and Allergies (2016). Dietary reference values for thiamin. EFSA J.

[25] EFSA Panel on Dietetic Products, Nutrition and Allergies (NDA) (2013). Scientific opinion on nutrient requirements and dietary intakes of infants and young children in the European Union. EFSA J.

[26] Hess S.Y., Smith T.J., Fischer P.R., Trehan I., Hiffler L., Arnold C.D. (2020). Establishing a case definition of thiamine responsive disorders among infants and young children in Lao PDR: protocol for a prospective cohort study. BMJ Open.

[27] Fumagalli M. (2020). Luang Prabang: climate change and rapid development. Cities.

[28] World Health Organization (2008).

[29] World Health Organization (2021).

[30] Cashin K., Oot L. (2018).

[31] WHO Multicentre Growth Reference Study Group (2006). methods and development.

[32] Lu J., Frank E.L. (2008). Rapid HPLC measurement of thiamine and its phosphate esters in whole blood. Clin. Chem..

[33] Jones K.S., Parkington D.A., Bourassa M.W., Cerami C., Koulman A. (2023). Protocol and application of basal erythrocyte transketolase activity to improve assessment of thiamine status. Ann. N. Y. Acad. Sci..

[34] Parkington D.A., Koulman A., Jones K.S. (2023). Protocol for measuring erythrocyte glutathione reductase activity coefficient to assess riboflavin status. STAR Protoc..

[35] Erhardt J.G., Estes J.E., Pfeiffer C.M., Biesalski H.K., Craft N.E. (2004). Combined measurement of ferritin, soluble transferrin receptor, retinol binding protein, and C-reactive protein by an inexpensive, sensitive, and simple sandwich enzyme-linked immunosorbent assay technique. J. Nutr..

[36] World Health Organization Western Pacific Region (2017). Guidelines for the management of common childhood illnesses.

[37] Hampel D., Shahab-Ferdows S., Islam M.M., Peerson J.M., Allen L.H. (2017). Vitamin concentrations in human milk vary with time within feed, circadian rhythm, and single-dose supplementation. J. Nutr..

[38] Hampel D., Shahab-Ferdows S., Adair L.S., Bentley M.E., Flax V.L., Jamieson D.J. (2016). Thiamin and riboflavin in human milk: effects of lipid-based nutrient supplementation and stage of lactation on vitamer secretion and contributions to total vitamin content. PLoS One.

[39] Hampel D., York E.R., Allen L.H. (2012). Ultra-performance liquid chromatography tandem mass-spectrometry (UPLC–MS/MS) for the rapid, simultaneous analysis of thiamin, riboflavin, flavin adenine dinucleotide, nicotinamide and pyridoxal in human milk. J. Chromotogr. B Analyt. Technol. Biomed. Life Sci..

[40] Schrijver J., Speek A.J., Klosse J.A., Van Rijn H.J.M., Schreurs W.H.P. (1982). A reliable semiautomated method for the determination of total thiamine in whole blood by the thiochrome method with high-performance liquid chromatography. Ann. Clin. Biochem..

[41] Ihara H., Hirano A., Wang L., Okada M., Hashizume N. (2005). Reference values for whole blood thiamine and thiamine phosphate esters in Japanese adults. J. Anal. Bio-Sci..

[42] Gibson R.S. (2005).

[43] Soukaloun D., Lee S.J., Chamberlain K., Taylor A.M., Mayxay M., Sisouk K. (2011). Erythrocyte transketolase activity, markers of cardiac dysfunction and the diagnosis of infantile beriberi. PLoS Negl. Trop. Dis..

[44] Allen L.H., Hampel D., Shahab-Ferdows S., Andersson M., Barros E., Doel A.M. (2021). The Mothers, Infants, and Lactation Quality (MILQ) study: a multi-center collaboration. Curr. Dev. Nutr..

[45] S.Y. Hess, T.J. Smith, C.D. Arnold. Lao thiamine study [Internet]. [cited 5 February, 2024]. 10.17605/OSF.IO/JFKE3.

[46] Perkins N.J., Schisterman E.F. (2006). The inconsistency of “optimal” cutpoints obtained using two criteria based on the receiver operating characteristic curve. Am. J. Epidemiol..

[47] FAO, FHI 360 (2016).

[48] Namaste S.M.L., Ou J., Williams A.M., Young M.F., Yu E.X., Suchdev P.S. (2019). Adjusting iron and vitamin A status in settings of inflammation: a sensitivity analysis of the Biomarkers Reflecting Inflammation and Nutritional Determinants of Anemia (BRINDA) approach. Am. J. Clin. Nutr..

[49] World Health Organization (2020).

[50] Wan Z., Zheng J., Zhu Z., Sang L., Zhu J., Luo S. (2022). Intermediate role of gut microbiota in vitamin B nutrition and its influences on human health. Front. Nutr..

[51] Porter S.G., Coats D., Fischer P.R., Ou K., Frank E.L., Sreang P. (2014). Thiamine deficiency and cardiac dysfunction in Cambodian infants. J. Pediatr..

[52] Nisar S., Yousuf Wani I., Altaf U., Muzaffer U., Kareem O., Tanvir M. (2024). Thiamine deficiency-related neuropathy: a reversible entity from an endemic area. Eur. J. Neurol..

[53] Nisar S., Mohi-U-Din K., Tak S.I., Andrabi S.M.A., Tanvir M., Muzaffer U. (2023). Thiamine responsive high output heart failure of adults: an under-recognized entity. Eur. J. Clin. Nutr..

[54] Gallant J., Chan K., Green T.J., Wieringa F.T., Leemaqz S., Ngik R. (2021). Low-dose thiamine supplementation of lactating Cambodian mothers improves human milk thiamine concentrations: a randomized controlled trial. Am. J. Clin. Nutr..

[55] Taylor A.J., Talwar D., Lee S.J., Cox L., Mayxay M., Newton P.N. (2020). Comparison of thiamin diphosphate high-performance liquid chromatography and erythrocyte transketolase assays for evaluating thiamin status in malaria patients without beriberi. Am. J. Trop. Med. Hyg..

[56] Floridi A., Pupita M., Palmerini C.A., Fini C., Alberti Fidanza A. (1984). Thiamine pyrophosphate determination in whole blood and erythrocytes by high performance liquid chromatography. Int. J. Vitam. Nutr. Res..

[57] Pfeiffer C.M., Fazili Z., Mineva E.M., Ngac P.K. (2021). First things first: a step in the right direction for the preanalytical phase of thiamine measurements. Am. J. Clin. Nutr..

