# Supplementary material for: Assessment of Erythrocyte Transketolase, Whole Blood Thiamine Diphosphate, and Human Milk Thiamine Concentrations to Identify Infants and Young Children Responding Favorably to Therapeutic Thiamine Administration: Findings from the Lao Thiamine Study, a Prospective Cohort Study
Source: Curr Dev Nutr. 2024 May 23;8(6):103786. doi: 10.1016/j.cdnut.2024.103786 (PMC11225667; doi:10.1016/j.cdnut.2024.103786)
Supplement: Multimedia component 1 [file mmc1.docx]

**Online supplementary material**

**Assessment of erythrocyte transketolase, whole blood thiamine diphosphate and human milk thiamine concentrations to identify children responding favorably to therapeutic thiamine administration: findings from the Lao Thiamine Study, a prospective cohort study**

Sonja Y Hess, Taryn J Smith, Charles D Arnold, Kerry S Jones, Daniela Hampel, Laurent Hiffler, Indi Trehan, Philip R Fischer, Sarah R Meadows, Damon A Parkington, Kenneth H Brown, Dalaphone Sitthideth, Xiuping Tan, Albert Koulman, Lindsay H Allen, Sengchanh Kounnavong

Page

| **Supplementary** **Table S1**: Eligibility criteria for study participants in the Lao Thiamine Study | 2 |
| --- | --- |
| **Supplementary** **Table S2:** TRD status among hospitalized children with at least one thiamine biomarker versus hospitalized children who were excluded from the present study due to thiamine administration prior to the initial blood draw | 4 |
| **Supplementary** **Figure S1:** Whole blood ThDP concentration, and basal ETK activity and ETKac among children with classic beriberi, probable TRD, possible TRD, not likely TRD and the frequency matched community comparison group and human milk concentration among their lactating mothers in the Lao Thiamine Study | 5 |
| **Supplementary** **Figure S2:** Association between whole blood ThDP concentration and ETKac among hospitalized children and the frequency matched community comparison group and their mothers in the Lao Thiamine Study | 7 |

**Supplementary** **Table S1**: Eligibility criteria for study participants in the Lao Thiamine Study

| Cohort and study participant | Target age range | Inclusion criteria | Exclusion criteria |
| --- | --- | --- | --- |
| Hospital  children | 21 days to  <18 months | - Liver enlargement (>2cm below right costal margin on supine exam while relaxed) - Edema - Tachypnea (> 60/min for 3-8 wks; > 50/min for 2-11 mo; > 40/min for 12-18 mo) - Tachycardia (heart rate >160/min for <12 mo; >120/min for 12-18 mo) - Oxygen saturation < 92% - Difficulty breathing (i.e. chest in-drawing, nasal flaring) - Refusal to breastfeed or refusal of infant formula or food for greater than 24 hours - Repetitive or recurring vomiting with no obvious other cause (i.e. vomiting >3 times in past 24 hours) - Persistent crying not relieved by soothing and feeding with no obvious other cause - Hoarse voice/cry or loss of voice - Nystagmus or other unusual eye movements - Muscle twitching - Loss of consciousness - Convulsion - Opisthotonus / abnormal posturing - Acute paralysis / flaccid paralysis   and Informed written consent by at least one parent or the primary caregiver | n/a |
| Hospital mothers | None | - Child enrolled in hospital cohort  - Informed written consent | - Severe acute illness warranting immediate hospital referral  - Unable to provide informed consent due to reduced decision-making ability |
| Community children | 21 days to  <18 months | - Meeting frequency matched characters (age, sex and location of residence) based on hospital cohort  - Informed written consent by at least one parent or the primary caregiver | - Severe acute illness warranting immediate hospital referral |
| Community mothers | None | - Child enrolled in community cohort  - Informed written consent | - Severe acute illness warranting immediate hospital referral  - Unable to provide informed consent due to reduced decision-making ability |

**Supplementary** **Table S2:** TRD status among hospitalized children with at least one thiamine biomarker versus hospitalized children who were excluded from the present study due to thiamine administration prior to the initial blood draw

| TRD Score^1^ | Children with ≥1 thiamine biomarker result | Children who received thiamine prior to blood draw^2^ |
| --- | --- | --- |
| N | 275^3^ | 136 |
| Classic beriberi | 50 (18.2) | 38 (27.9) |
| Probable TRD | 103 (37.5) | 56 (41.2) |
| Possible TRD | 102 (37.1) | 40 (29.4) |
| Not likely | 20 (7.3) | 2 (1.5) |

TRD, thiamine responsive disorders

Results shown as n (%)

^1^ Three expert pediatricians independently reviewed the case reports of each hospitalized child to judge whether a child had TRD using a four-point scale (classic beriberi; probable TRD; possible TRD and not likely TRD). The categories classic beriberi and probable TRD were subsequently combined to ‘TRD’; and possible TRD and not likely TRD were combined as ‘non-TRD’.

^2^ We excluded thiamine biomarkers for women and children who reportedly received supplementation prior to blood draw or had a free thiamine concentration greater than the 90^th^ percentile of the study sample. Of the 146 participants for which this was necessary, 10 were participants who did not have a TRD diagnosis.

^3^ Of the 287 hospitalized children with ≥1 thiamine biomarker result, 12 do not have a TRD diagnosis, resulting in a final n=275 for children with biomarker results and TRD status.

**Supplementary** **Figure S1:** Whole blood ThDP concentration, and basal ETK activity and ETKac among children with classic beriberi, probable TRD, possible TRD, not likely TRD and the frequency matched community comparison group and human milk thiamine (MTh) concentration among their breastfeeding mothers in the Lao Thiamine Study^1^

ETK, erythrocyte transketolase; ETKac, erythrocyte transketolase activity coefficient; MTh, human milk thiamine; ThDP, thiamine diphosphate; TRD, thiamine responsive disorders.

^1^The lower and upper borders of the box are the 25^th^ and 75^th^ percentiles of the distribution, while the line within the box is the median. The whisker extends to the values that are furthest away from the median while still within 1.5 times the interquartile range. Outside points are values outside this range.

**Supplementary** **Figure 2**: Association between whole blood ThDP concentration and ETKac among hospitalized children and the frequency matched community comparison group and their mothers in the Lao Thiamine Study^1^

ETKac, erythrocyte transketolase activity coefficient; ThDP, thiamine diphosphate

^1^Spearman correlations used for analysis and values log transformed for regression analyses.
